# Network assisted analysis of *de novo* variants using protein-protein interaction information identified 46 candidate genes for congenital heart disease

**DOI:** 10.1371/journal.pgen.1010252

**Published:** 2022-06-07

**Authors:** Yuhan Xie, Wei Jiang, Weilai Dong, Hongyu Li, Sheng Chih Jin, Martina Brueckner, Hongyu Zhao

**Affiliations:** 1 Department of Biostatistics, Yale School of Public Health, New Haven, Connecticut, United States of America; 2 Department of Genetics, Yale School of Medicine, New Haven, Connecticut, United States of America; 3 Department of Genetics, Washington University School of Medicine, St Louis, Missouri, United States of America; 4 Department of Pediatrics, Yale University, New Haven, Connecticut, United States of America; 5 Program in Computational Biology and Bioinformatics, Yale University, New Haven, Connecticut, United States of America; Case Western Reserve University, UNITED STATES

## Abstract

*De novo* variants (DNVs) with deleterious effects have proved informative in identifying risk genes for early-onset diseases such as congenital heart disease (CHD). A number of statistical methods have been proposed for family-based studies or case/control studies to identify risk genes by screening genes with more DNVs than expected by chance in Whole Exome Sequencing (WES) studies. However, the statistical power is still limited for cohorts with thousands of subjects. Under the hypothesis that connected genes in protein-protein interaction (PPI) networks are more likely to share similar disease association status, we developed a Markov Random Field model that can leverage information from publicly available PPI databases to increase power in identifying risk genes. We identified 46 candidate genes with at least 1 DNV in the CHD study cohort, including 18 known human CHD genes and 35 highly expressed genes in mouse developing heart. Our results may shed new insight on the shared protein functionality among risk genes for CHD.

## Author summary

The topologic information in a pathway may be informative to identify functionally interrelated genes and help improve statistical power in DNV studies. Under the hypothesis that connected genes in PPI networks are more likely to share similar disease association status, we developed a novel statistical model that can leverage information from publicly available PPI databases. Through simulation studies under multiple settings, we proved our method can increase statistical power in identifying additional risk genes compared to methods without using the PPI network information. We then applied our method to a real example for CHD DNV data, and then visualized the subnetwork of candidate genes to find potential functional gene clusters for CHD.

## Introduction

Congenital heart disease (CHD) is the most common birth defect affecting ~ 1% of live births and accounts for one-third of all major congenital abnormalities [1–3]. There is substantial evidence that CHD has a strong genetic component [4]. Although it is estimated that aneuploidies and copy number variations account for about 23% of CHD cases, few individual disease-causing genes have been identified in published studies [5–8]. Therefore, the limited knowledge of the underlying genetic causes poses an obstacle to the reproductive counseling of CHD patients [9].

Whole Exome Sequencing (WES) studies have successfully boosted novel causal gene identification for both Mendelian and complex disorders [10,11]. To narrow down the pool of candidate variants from WES, family-based studies have been conducted to scan for *de novo* variants (DNVs) from parent-offspring trios. DNV studies have been shown to play an important role in risk gene identification for CHD [1,3,5,6,12–15]. From the analysis of 1,213 CHD parent-offspring trios, Homsy et al. identified a greater burden of damaging DNVs, especially in genes with likely functional roles in heart and brain development [12]. Recently, Jin et al. inferred that DNVs in ~440 genes were likely contributors to CHD [5]. Despite these advances, it remains challenging to capture the causal genes with only DNV data as CHD is very genetically heterogeneous [6].

Several statistical methods have been proposed to identify risk genes by integrating DNVs with other genetic variants and additional biological data. He et al. developed a Bayesian framework, namely the Transmission And De novo Association (TADA), to increase statistical power of inferring risk genes by incorporating both DNMs and rare inherited variants [16]. A hierarchical Bayes strategy was adopted for parameter estimation in TADA. Following this idea, a number of methods have been proposed to improve TADA, with some focusing on leveraging the shared genetic information in multiple correlated phenotypes, such as neurodevelopmental disorders and CHD [17,18], whereas others extend the method by integrating DNMs with other types of genetic variants and functional annotations [19–22]. Please note that, except for DECO [22], all these methods treat each gene individually and do not consider the interaction effects of genes. Thus, there is a pressing need for developing network-based frameworks to consider the functional connectivities among genes.

Network-based approaches have been successful in prioritizing risk genes for downstream genomic and transcriptomic studies [23–26]. Chen et al. [24] proposed a Markov Random Field (MRF) model to incorporate pathway topology structure for Genome-Wide Association Studies (GWAS). They showed that their method is more powerful than single gene-based methods through both simulation and real data analyses. In 2015, Liu et al. adopted a similar idea as Chen et al. to analyze DNV data from WES studies [27]. Their framework, namely DAWN, combines TADA p-values with the estimated network from gene co-expression data. In their real data analysis for autism, 333 genes were prioritized by integrating DNV summary statistics and expression data from brain tissue. However, the above methods require summary statistics (Z scores or p-values) from genetic association analysis as their input, which may not be provided from results of DNV analysis [17,19].

More recently, Bayrak et al. developed a priority score to quantify the proximity of genes to the known CHD risk genes using DNV data [3]. Utilizing canonical pathways and human gene networks, their analyses identified 23 novel genes that are likely to contribute to CHD pathogenesis. Their results further support the potential to improve power by integrating network information with DNV data. Then, the question becomes how to choose an informative gene network for CHD. As there is a limited number of co-expression data sets for human developmental heart, a natural choice for network information would be human PPI databases. There are multiple primary PPI network databases such as BioGRID [28], IntAct [29], DIP [30], MINT [31], and HPRD [32]. Most network-based studies apply their real data on two or more of databases to obtain their results. Nonetheless, it is hard to check the overlapping information between two PPI databases and interpret the divergent results. Multiple integrative databases such as STRING [33], HINT [34], UniHI [35], hPRINT [36] and GPS-Prot [37] provide a platform to resolve the above problems [38]. Among them, STRING is a popular PPI resource that imports protein association knowledge from physical interaction and curated knowledge from the primary PPI databases and other pathway information knowledge such as KEGG [39–41] and GO [42,43]. In addition, it provides a score to measure the likelihood of interactions. Some studies have used STRING in their post-association analysis for gene-based DNV studies and showed significant enrichment of candidate CHD risk genes in the STRING PPI network [44,45]. These results suggest that incorporating PPI network information from STRING may identify additional risk genes with more biological interpretability.

**Fig 1:**
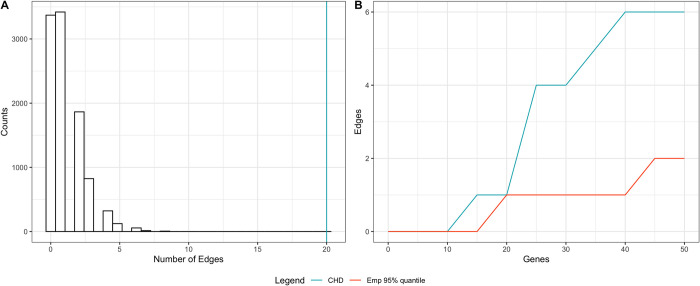
CHD top genes are more connected than randomly selected genes in the STRING PPI network. (A) Empirical distribution of the number of edges formed by 21 randomly selected genes. Blue line represents the number of edges formed by the 21 CHD top genes from TADA *de novo* analysis. (B) Blue line represents the number of edges formed by CHD top genes and red line represents randomly selected genes.

As an illustrative example, we applied TADA *de novo* test [16] with the CHD DNV data curated in our previous work [18], and conducted a post-association analysis on the p-values returned from the test. After false discovery rate (FDR) adjustment of p-values, we identified 21 genes with FDR<0.1 among 18,856 genes tested, and found that the number of edges formed by the 21 genes (20 edges, blue line in Fig 1A) is much larger than the upper tail of the empirical distribution sampled from 21 randomly selected genes in the STRING V11.0 database (score threshold: 400) for 10,000 times (Fig 1A). This suggests that the candidate CHD genes are highly enriched in terms of their interactions in the STRING database. To further illustrate that PPI information may contribute to CHD gene discovery, we showed the number of edges formed by the top genes ranked by adjusted p-values for CHD and compared it with the number of edges formed by randomly selected genes with a more stringent selection of PPI edges in the STRING database (score threshold: 950) in Fig 1B. We considered 95^th^ percentile of the empirical distribution derived from 10,000 sets of random genes in the PPI network as a baseline. When more than 20 top CHD genes are selected, the number of edges formed by these genes is significantly more than that from randomly selected genes. This suggests top genes in CHD tend to be neighbors in the STRING PPI network.

Motivated by the observation from Fig 1, we develop a **N**etwork assisted model for **D**e novo **A**ssociation **T**est using protein-protein inter**A**ction information, named N-DATA, to leverage prior information of interactions among genes from the PPI network to boost statistical power in identifying risk genes for CHD based on the ‘guilt by association’ principle [46, 47]. In the following, we first introduce the inference procedure for our model, and then demonstrate the performance of our method through simulation studies and real data applications.

## Methods

In this section, we introduce the statistical model for the proposed framework. The network information in the PPI database is represented by an undirected graph *G* = (*V*,*E*), where *V* = {1,..,*n*} is a set of *n* genes in the network, and *E* = {<*i*,*j*>: *i* and *j* are genes connected by the edges}. The degree of a gene *i* is defined as the number of direct neighbors (*N*_*i*_) for gene *i* in the network and denoted as *d*_*i*_. We denote the latent association status of gene *i* with a disease of interest, e.g., CHD, as *S*_*i*_, where *S*_*i*_ = 1 if gene *i* is associated with the disease, *S*_*i*_ = −1 if gene *i* is not associated with the disease. *S* = {*S*_1_,..,*S*_*n*_} are the corresponding latent states for genes in *V* = {1,…,*n*}. The DNV count of each gene *i* is defined as *Y*_*i*_. We propose a simple discrete Markov random field model [48, 49] with a nearest neighbor Gibbs measure [50] to model the following joint probability function *S* = {*S*_1_,..,*S*_*n*_}:

$$P(S|\theta_{0})\propto \exp\{h\sum_{i\in V}I_{1}(S_{i})+\tau_{0}\sum_{<i,j>\in E}\left(w_{i}+w_{j})I_{-1}(S_{i})I_{-1}(S_{j}\right)+\tau_{1}\sum_{<i,j>\in E}\left(w_{i}+w_{j})I_{1}(S_{i})I_{1}(S_{j}\right)\},$$

where *w*_*i*_ is the weight for gene *i* and will be chosen based on the characteristics of the network. In real data analysis, we set *w*_*i*_ as the square root of the degree of gene *i* in the network ($w_{i}=\sqrt{d_{i}}$) following Chen et al. [24]. *θ*_0_ = (*h*, *τ*_0_, *τ*_1_) are hyperparameters related to the network. Specifically, *h* determines the marginal distribution of *S*_*i*_ when all genes are independent i.e., $P(S_{i}=1|h,\tau_{0}=\tau_{1}=0)=\frac{\exp\left(h\right)}{1+\exp\left(h\right)}$. *τ*_0_ and *τ*_1_ characterize the prior weights of edges between non-associated genes and associated genes, respectively. We further assume that, given the latent state *S*_*i*_, the DNV count *Y*_*i*_ follows a Poisson distribution. The mutability of gene *i* (*μ*_*i*_) can be estimated using the framework in Samocha et al. [12, 51]. Based on the derivation in TADA [16], the probability of observing DNVs for gene *i* in each trio can be approximated by 2*μ*_*i*_*γ*, where *γ* is the relative risk of the DNVs. Further, the expected count of DNVs for gene *i* in N trios is 2*Nμ*_*i*_*γ*. When gene *i* is not a risk gene, *γ* is equal to 1. Then, we have the following model for DNV counts:

$$Y_{i}|S_{i}=-1∼Poisson(2N\mu_{i}),$$

$$Y_{i}|S_{i}=1∼Poisson(2N\mu_{i}\gamma ),$$

$$\theta_{0}=(h,\tau_{0},\tau_{1});\theta_{1}=\gamma .$$

To reduce the computational burden from a fully Bayesian solution for maximizing the marginal likelihood, we propose an empirical Bayes method to estimate the parameters *θ*_0_ and *θ*_1_, and the latent association status *S* by maximizing the pseudo conditional likelihood (PCLK) for *n* genes as follows

$$PCLK=\prod_{i=1}^{n}f(Y_{i}|S_{i},\theta_{1})\mathrm{Pr}\left(S_{i}|S_{N_{i}},\theta_{0}\right),$$

where $S_{N_{i}}$ represents the latent association status for neighbors of gene *i*. It has been shown that the estimator from the PCLK in a general Markov random field setting is consistent under mild regularity conditions [24,49]. When maximizing the PCLK, we can estimate the hyperparameters *θ*_0_, *θ*_1_ and latent status *S* iteratively.

We can obtain an empirical estimate for *θ*_0_ by maximizing $\prod_{i=1}^{n}\mathrm{Pr}\left(S_{i}|S_{N_{i}},\theta_{0}\right)$, which is equivalent to maximizing the parameters in the following logistic regression model:

$$\mathrm{logit}\;\mathrm{Pr}(S_{i}|S_{N_{i}},\theta_{0})=h+\tau_{1}X_{i1}-\tau_{0}X_{i0},$$

where $X_{i1}=w_{i}\sum_{k\in N_{i}}I_{1}(S_{k})+\sum_{k\in N_{i}}w_{k}I_{1}(S_{k})$ and $X_{i0}=w_{i}\sum_{k\in N_{i}}I_{-1}(S_{k})+\sum_{k\in N_{i}}w_{k}I_{-1}(S_{k}),i=1,\ldots ,n$. To make sure the estimated *θ*_0_ is finite, we can add a ridge penalty term $\lambda (h^{2}+\tau_{0}^{2}+\tau_{1}^{2})$ to the likelihood function to solve the maximization problem by the Newton-Raphson’s method [52].

We then update the latent status *S* by maximizing the PCLK using the iterative conditional mode method [49]. After we obtain the updated values *θ*_0_ and *S*, we can estimate the hyperparameter *θ*_0_ by maximizing $\prod_{i=1}^{n}f(Y_{i}|S_{i},\theta_{1})$ by using the following closed-form expression:

$$\log\;L(\theta_{1}|Y)\propto \log\prod_{S_{i}=1}\exp\left(-2\mu N\gamma +Y_{i}\log\gamma \right)$$

$$\frac{\partial \log\;L(\theta_{1}|Y)}{\partial \gamma }=-\sum_{S_{i}=1}2\mu N+\frac{\sum_{S_{i}=1}Y_{i}}{\gamma }$$

$$\hat{\gamma }=\frac{\sum_{S_{i}=1}Y_{i}}{\sum_{S_{i}=1}2\mu N}$$

Algorithm 1: Procedure for Parameter Estimation

1. Set initial configuration ***S***^0^

2. In the *j*th iteration, for given ***s***^(*j*−1)^, obtain ${\hat{\theta }}_{0}^{j}$ from

$$logit\;Pr\;(S_{i}^{\left(j-1\right)}|S_{N_{i}}^{\left(j-1\right)},\theta_{0}^{\left(j-1\right)})=h+\tau_{1}X_{i1}-\tau_{0}X_{i0},\;i=1,\cdots ,n$$

3. Sequentially update the labels of nodes to obtain *S*^(*j*)^ (ICM)

Si(j)=argmaxsif(Yi|Si,θ^1(j−1))Pr(Si|SNi(j−1),θ^0(j))∏k∈SNPr(Sk(j−1)|Si,SNk−i(j−1),θ^0(j))

4. Obtain ${\hat{\theta }}_{1}^{j}({\hat{\gamma }}^{\left(j\right)})$ from

$${{\hat{\theta }}_{1}}^{\left(j\right)}={argmax}_{\theta_{1}}\;log\;L(\theta_{1}|\theta_{0}^{\left(j\right)},S^{\left(j\right)},Y)$$

5. Repeat steps 2, 3, and 4 until convergence

Finally, after we obtain the estimated ${\hat{\theta }}_{0}$ and ${\hat{\theta }}_{1}$, we use Gibbs sampling based on the conditional distribution $P(S_{i}|S_{N_{i}},{\hat{\theta }}_{0},{\hat{\theta }}_{1})$. This method has been proved to be valid for multiple testing under dependence in a compound decision theoretic framework [53,54]. Then, we can estimate the marginal posterior probability *q*_*i*_ = *P*(*S*_*i*_ = −1|*Y*). Let *q*_(*i*)_ be the sorted values of *q*_*i*_ in descending order. For each gene *i*, the null hypothesis and alternative hypothesis are

$$H_{i0}:\;\mathrm{Gene}\;i\;\mathrm{is}\;\mathrm{not}\;\mathrm{associated}\;\mathrm{with}\;\mathrm{the}\;\mathrm{trait}\;\mathrm{of}\;\mathrm{interest}$$

$$H_{i1}:\;\mathrm{Gene}\;i\;\mathrm{is}\;\mathrm{associated}\;\mathrm{with}\;\mathrm{the}\;\mathrm{trait}\;\mathrm{of}\;\mathrm{interest}$$

As shown by Jiang and Yu [55], the relationship between global FDR and local FDR (lfdr) is FDR = *E*(lfdr|*Y*∈ℛ), where the rejection region ℛ is the set of *Y* such that the null hypothesis can be rejected based on a specific rejection criterion. To control the expected global FDR less than *α*, we propose the following procedure: let $m=max\;\{s:\frac{1}{s}\sum_{i=1}^{s}q_{\left(i\right)}\}$, we reject all the null hypotheses corresponding to *H*_(1)_,…,*H*_(*m*)_.

### Verification and comparison

We used network information from the STRING PPI database and simulated DNV count data to study the performance of our method. First, we randomly selected 2,000 genes, retrieved their mutability from the real data, and extracted the corresponding PPI network formed by these 2,000 genes. Then, we simulated the latent status of genes with Gibbs sampling under the given network information, and the count of DNVs for each gene with the Poisson distribution given the latent status of the gene. We evaluated FDR and power under various settings of sample size *N* and relative risk parameter *γ*.

We fixed true network parameter *h* as -4 and varied *τ*_1_ from 0.1 to 0.9 to make the total number of risk genes in the network of 2,000 random genes vary from 57 to 353. We varied the sample size *N* at 2,000, 5,000 and 10,000 to evaluate the performance of N-DATA in small, medium, and large WES cohorts, respectively. In addition, we varied *β* (log relative risk parameter *γ*) at 3, 3.5, and 4 to investigate the performance of N-DATA around the burden estimated results from real data (In real data analysis, $\hat{\beta }$ = 3.60). Each simulation setting was replicated 100 times. For Gibbs sampling-based inference, we used 5,000 MCMC iterations, and set the first 2,000 iterations as burn-ins. These numbers were chosen empirically based on the diagnostic plots for convergence.

First, we compared the performance of N-DATA model with and without the PPI network as input. For N-DATA model without the PPI network, we assigned the weight of gene *i w*_*i*_ = 0 for inference. We present the power and FDR performance of N-DATA models in Fig A and Fig B in S1 Text. Then, we compared the power of TADA *de novo* test (TADA-*De novo*), DAWN, and N-DATA using the same simulation settings. Hyperprior of TADA-*De novo* was estimated from the function *denovo*.*MOM* based on the recommendation from the authors [16]. Power of TADA was calculated based on TADA p-values under FDR adjustment. DAWN v1.0 was downloaded from http://www.compgen.pitt.edu/DAWN/DAWN_homepage.htm. We adapted the code of DAWN by substituting the adjacency matrix inferred from its Partial Neighborhood Selection algorithm to the adjacency matrix from network. We used TADA-*De novo* p-values and PPI network as the input of DAWN. We applied default settings for parameters in DAWN.

**Fig 2:**
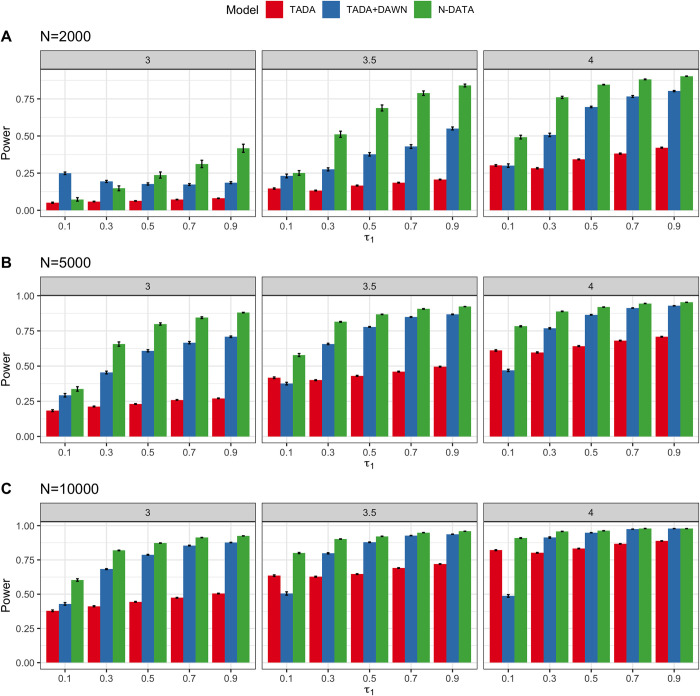
Power comparison of TADA-*De novo*, TADA-*De novo* p-values + DAWN and N-DATA. Error bars represent standard errors estimated from 100 replications of simulation. Three panels in each sub-figure from left to right represent *β* = 3, *β* = 3.5, and *β* = 4, respectively. Each panel shows the change of power when *τ*_1_ varies from 0.1 to 0.9. (A) Power comparison between the two models when the sample size is small (*N* = 2,000). (B) Power comparison between the three models when the sample size is medium (*N* = 5,000). (C) Power comparison between the three models when the sample size is large (*N* = 10,000).

We compared the performance of TADA-*De novo*, TADA-*De novo* p-values + DAWN and N-DATA under different simulation settings. We reported the power performance under FDR threshold 0.05 in the main text (Fig 2). We first checked if all three methods could control the global FDR when the threshold is 0.05 (Fig C in S1 Text). Overall, N-DATA controlled the FDR well and had the best power under all scenarios. We observed that when *τ*_1_, *N*, and *β* are all small, DAWN had FDR inflations for some runs. We suspect that this may be due to the discreteness of p-values, resulting in the violation of the normal distribution assumption for corresponding z-scores used in the input of DAWN. When the number of risk genes is small, DAWN may have lower power than TADA and N-DATA. When *τ*_1_, *N*, and *β* became larger, the power of DAWN was comparable with N-DATA. Time comparisons for the three models are presented in Fig D in S1 Text.

### Application

We applied N-DATA to DNV data from 2,645 CHD trios reported in Jin et al [5], and annotated the CHD variants by ANNOVAR [56]. We denoted loss of function (LoF) as frameshift insertion/deletion, splice site alteration, stopgain and stoploss predicted by ANNOVAR, and deleterious missense (Dmis) predicted by the MetaSVM [57] algorithm. We only consider damaging variants (LoF and Dmis) in our analysis as the number of non-deleterious variants is not expected to provide the information to differentiate cases from controls biologically [58].

For network information, we first downloaded STRING v11.0 with medium edge likelihood via interface from STRINGdb package in R and call this original network from STRING $G_{0}$. We obtained the curated list of known human CHD genes from Jin el al [5] and expanded the gene list by including additional candidate genes (FDR<0.1) from the single-trait analysis in our previous work [18]. This gene list (258 genes) was set as seed genes for our network. Then, we extracted the subnetwork including the seed genes and the direct neighbors with likelihood score larger than 950 of those genes and call this subnetwork $G_{1}$. We only kept overlapping genes with our DNV data in $G_{1}$ and called the final network used in our real application as $G_{2}$. There were in total 1,814 genes and 21,468 edges in $G_{2}$.

To show that our method can leverage network information to boost risk gene identification, we applied our algorithm without using the network as an input. When there was no prior information from the network, we identified 18 significant genes with FDR<0.05. To include the network information from $G_{2}$we denote the degree of gene *i* in network $G_{2}$ as *d*_*i*_, and let the weight in the prior as $w_{i}=\sqrt{d_{i}}$. After adding the network information from $G_{2}$, we identified 46 genes with at least 1 DNV, and 26 genes harboring at least 2 DNVs with FDR<0.05 in the CHD cohort.

We also compared the results of N-DATA with TADA-*De novo* test [16]. As in the simulation study, we observed that DAWN may not control the FDR under the preset threshold under our network and cohort settings. Thus, we did not include the results of DAWN in the comparison. TADA-*De novo* test (p-values with FDR adjustment) identified 28 significant genes. Without integrating the network information, N-DATA can identify 18 significant genes with FDR<0.05. After integrating the $G_{2}$ network, N-DATA identified 323 genes with FDR<0.05. As some of the genes may be prioritized due to network characteristics, but did not have DNV count in the study cohort (more details in S1 Text), we further filtered out genes without DNV and considered the 46 genes identified with FDR<0.05 and at least 1 DNV as the candidate genes. (Table 1)

**Table 1:** Comparison of TADA and N-DATA models.

Method	Criteria	Number of Identified Genes
TADA-*De novo* p-values	FDR<0.05	28
N-DATA w/o Network	FDR<0.05	18
N-DATA (Network $G_{2}$ network + DNV counts)	FDR<0.05 DNVs≥1	323 46

**Fig 3:**
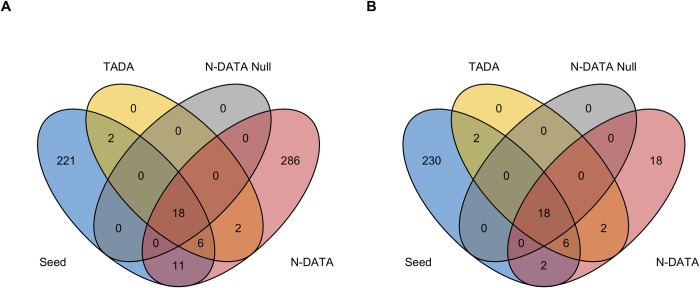
Venn diagram of 258 seed genes, TADA genes, N-DATA w/o network genes and N-DATA genes. (A) Overlapping genes between 258 seed genes, TADA genes, N-DATA w/o network (N-DATA Null) genes and 323 N-DATA genes. (B) Overlapping genes between 258 seed genes, TADA genes, N-DATA w/o network (N-DATA Null) genes and 46 N-DATA candidate genes.

We visualized the overlap of 258 seed genes, genes that were identified by TADA-*De novo* p-values, N-DATA w/o network model, and N-DATA in Fig 3. Fig 3A shows the 323 genes identified by N-DATA, while Fig 3B shows the 46 genes with at least 1 DNV. From Fig 3B, N-DATA found most of the genes that can be identified by TADA (26 out of 28).

**Fig 4:**
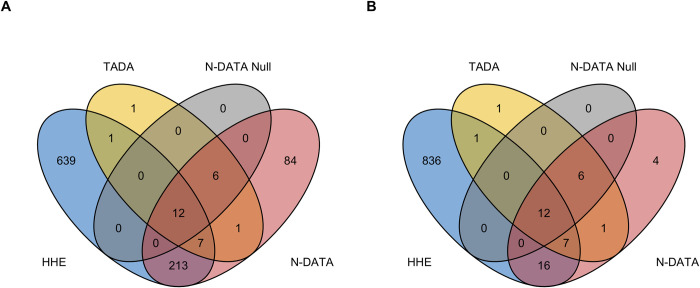
Venn diagram of HHE genes, TADA genes, N-DATA w/o network genes, and the N-DATA genes. (A) Overlapping genes between 872 HHE genes, TADA genes, N-DATA w/o network (N-DATA Null) genes, and 323 N-DATA genes. (B) Overlapping genes between 872 HHE genes, TADA genes, N-DATA w/o network (N-DATA Null) genes, and 46 N-DATA candidate genes.

Further, we calculated the overlap of the significant genes identified by N-DATA and TADA, and 872 genes that are highly expressed (top 25%) in mouse developing heart at E14.5 [12] and in the 1,814 gene network (HHE genes) (Fig 4). Among the 323 N-DATA identified genes, 27 are known human CHD genes and 213 genes are HHE genes. Among the 46 genes, 18 are known human CHD genes and 35 are HHE genes.

We visualized the 323 genes identified in the $G_{2}$ network (S1 Fig). The 323 genes formed two major clusters. The bigger cluster (right) is an extended cluster for protein synthesis genes, including ribosome protein genes (RPL-, RPS-), peptide chain elongation genes (EEF-, EIF-, SPR-, GSPT-), rRNA processing genes (UTP-, WDR-, RIOK-, NO-, IMP-), etc. Though without finding DNVs in the current cohort, ribosome genes *RPL11*, *RPL35A*, *RPS10*, *RPS19*, *RPS24*, *RPS26*, and *RPS7* are known CHD genes. Ribosome dysfunctions have been implicated in a variety of developmental disorders, including CHD [59]. For instance, multiple genes encoding ribosome subunits are known to cause Diamond-Blackfan anemia and 30% of the patients also presented CHD [60]. Functional studies showed that the deficiency in ribosomes can impact cell growth which might be a potential mechanism to cause CHD [61,62].

The other cluster (left) is the extended cluster for mRNA splicing genes, which encode various components of spliceosome and associated factors, such as snRNP (LSM-, SNRNP-, SNRP-), pre-mRNA processing factors (PRPF-), RNA helicases (DDX-, DHX-), hnRNPs (HNRNP-), and splicing factors (SF3-, SRS-, CWC-) [63]. Heart development involves many alternative splicing events. Mutations in splicing associated factor genes such as *RBM24*, *RBFOX2* and *SF3B1*, have been shown to cause cardiac malformation in mouse and human [64]. A specific type of snRNP called snoRNA and its targets showed reduced expression in myocardium of infants with Tetralogy of Fallot and impacted heart development through impairing spliceosome functions [65].

Thus, genes in the two clusters may be associated with CHD via disruption of protein synthesis or mRNA splicing events.

**Fig 5:**
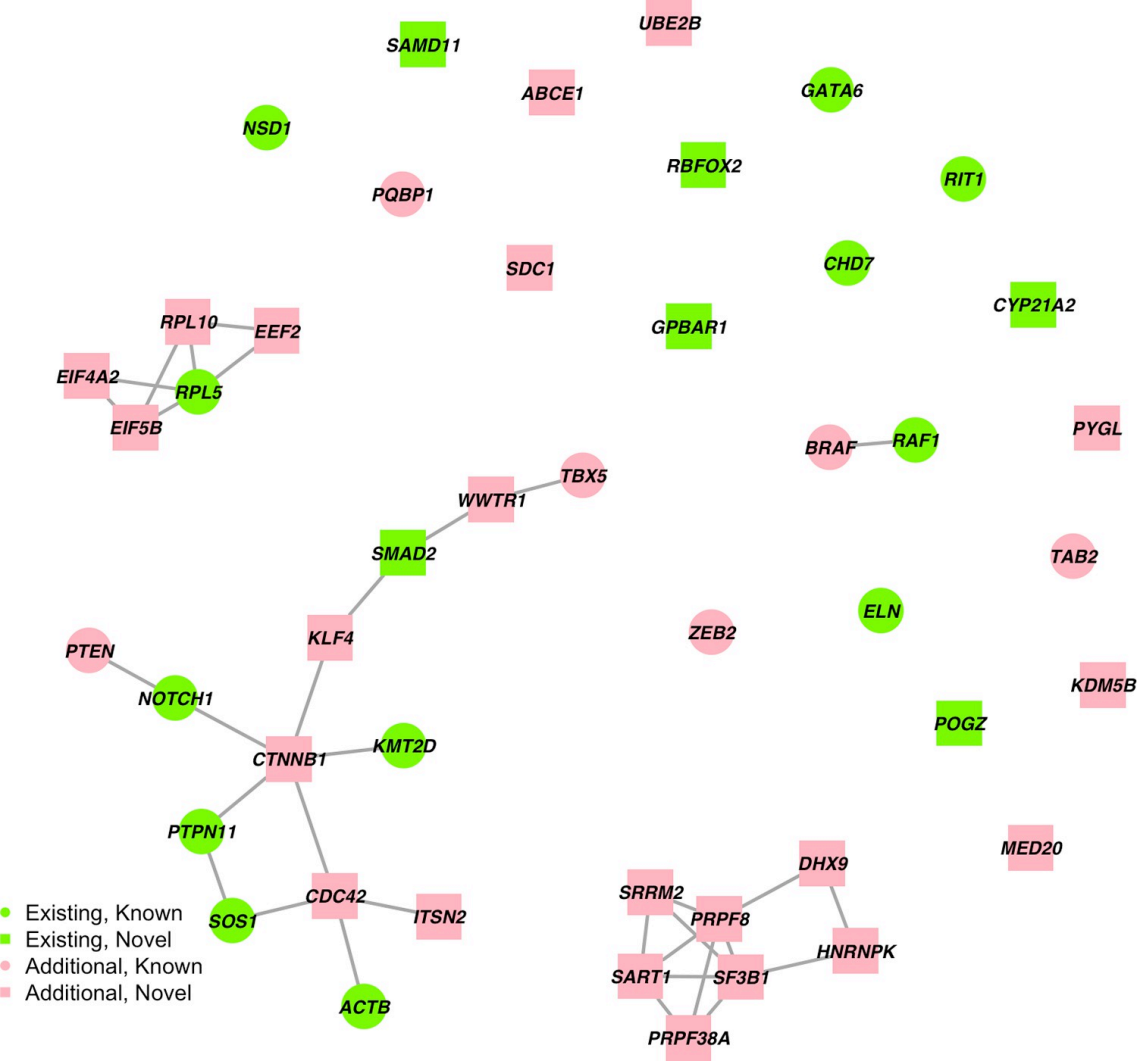
N-DATA model identified 46 candidate genes with at least 1 DNV. Green labels indicate the 18 genes identified when no network information was provided for N-DATA, and red labels indicate the additional 28 genes identified when the $G_{2}$ network was integrated. Circles indicate the 18 known human CHD genes, and squares indicate the 28 novel genes identified by N-DATA.

Further, we zoomed in on the 46 genes with at least 1 DNV in the $G_{2}$ network to demonstrate that the PPI network information can help boost statistical power and provide biological interpretation for the current CHD cohort. (Fig 5)

Among the 46 candidate genes, *PTPN11*, *RAF1* and *RIT1* had 2 recurrent DNVs, and *CHD7*, *NOTCH1*, *NSD1* and *PYGL* also had recessive genotypes in the CHD cohort [5]. The 46 candidate genes form 4 clusters in the $G_{2}$ network (Fig 5). The biggest cluster includes seven known CHD genes *TBX5*, *KMT2D*, *PTPN11*, *SOS1*, *ACTB*, *NOTCH1*, and *PTEN*, which are involved in transcriptional regulation and early cell growth or differentiation processes. The six new genes *SMAD2*, *KLF4*, *CTNNB1*, *CDC42*, *ITSN2*, and *WWTR1* also function in similar pathways and have varied implications for cardiac development. For instance, *KLF4* and *CTNNB1* have been implicated in cardiac cell differentiation [66]. *Cdc42* cardiomyocyte knock-out mice presented heart defects such as ventricular septum defects and thin ventricular walls [67]. *WWTR1* encodes a transcription regulator, which serves as an effector of Hippo pathway and regulates cardiac wall maturation in zebrafish [68].

The second biggest cluster is constituted of 7 new genes, all of which are involved in mRNA splicing. Specifically, *SART1*, *SRRM2*, *PRPF38A*, *PRPF8*, and *SF3B1* are associated factors or components of spliceosome; *HNRNPK* encodes a pre-mRNA-binding protein; *DHX9* encodes an RNA helicase which promotes R-loop formation while RNA splicing is perturbed [69]. Alternative splicing plays an essential role in heart development, homeostasis, and disease pathogenesis. Mouse knockouts of multiple splice factors had impaired cardiogenesis [70]. *SF3B1*, specifically, has been shown to upregulate to induce heart disease in both human and mice [64]. Thus, though not fully investigated, DNVs in those mRNA splicing-related genes may contribute to CHD pathogenesis.

The third cluster contains genes involved in protein synthesis, including the known gene *RPL5* and genes not previously associated with CHD (*EIF4*, *EIF*5, *EEF2*, and *RPL10)*. *RPL5* and *RPL10* encode the ribosome subunits. Mutations in *RPL5* and other ribosomal genes can lead to multiple congenital anomalies, including CHD [71]. *EIF4* and *EIF5* encode translation initiation factors while *EEF2* encodes the elongation factor that regulate peptide chain elongation during protein synthesis. A recent study reported that the deficiency in ribosome associated NatA complex reduces ribosomal protein and subsequently impact cell development as a mechanism to cause CHD [62]. Thus, DNVs in the above genes may lead to CHD via impairment of protein synthesis.

The last cluster contains the known CHD genes *BRAF* and *RAF1*, both of which encode key kinases in Ras signaling and are related to Noonan syndrome with CHD as a common feature.

Among the un-clustered genes, six are identified after using the network information: *ABCE1*, *UBE2B*, *SDC1*, *PYGL*, *KDM5B*, *MED20*. *UBE2B* and *KDM5B*, encoding epigenetic modifiers, have shown suggestive evidence in cardiac development or CHD [72,73] and might be potential CHD genes.

## Discussion

In this article, we have introduced a Bayesian framework to integrate PPI network information as the prior knowledge into DNV analysis for CHD. The implemented model is available at https://github.com/JustinaXie/NDATA. This approach adopts MRF to model the interactions among genes. We apply an empirical Bayes strategy to estimate parameters in the model and conduct statistical inference based on the posterior distribution sampled from a Gibbs sampler. The simulation studies and real data analysis on CHD suggest that the proposed method has improved power to identify risk genes over methods without integrating network information.

Our proposed framework is innovative in the following aspects. First, it does not need to estimate hyperprior based on other sources compared to the existing pathway-based test for DNV data [22,45]. Second, it does not require external expression data for the DNV cohort and uses the publicly available PPI database instead, which makes it more applicable to different diseases. This method not only increases power in risk gene identification, but also assists in biological interpretation by visualizing clusters of risk genes with functional relevance in the network.

However, there are some limitations in the current N-DATA model. First, our model is dependent on the choice of network. Using different PPI networks and different filtering criteria could result in a different set of significant genes. Currently, we did not provide a way to prioritize existing networks. We have provided details on a comparison of using the HINT network versus the STRING network (more details in S1 Text). For the two networks compared in our study, HINT has the advantage of leveraging additional information from PDB [74], and being manually curated to filter out erroneous and low-quality interactions; while STRING has the advantage of providing a score to measure the likelihood of interactions, and including information from multiple pathway databases. We also found that the risk genes identified from the two databases had a significant overlap (*p* = 3.14×10^−12^). The overlapping risk genes were highly enriched for Human Phenotype Ontology (HPO) [75] terms related to CHD from g:Profiler [76] analysis, and p-values of overlapped pathway outputs from Ingenuity Pathways Analysis (IPA, QIAGEN Inc.) had a significant Pearson’s correlation (*R* = 0.48; *p*<2.2×10^−16^).

Second, our model may be only used for early on-set disorders with a strong DNV signal. For diseases with small relative risks or small sample sizes, our model may suffer from convergency issues (more details in S1 Text). In real applications, it is important to conduct an initial analysis on the enrichment of top genes identified from *de novo* association tests in the network like our motivating example.

Third, we applied an empirical Bayes strategy to obtain point estimates of hyperparameters instead of using a fully Bayesian approach considering the computation burden. A fully Bayesian model that can account for intrinsic uncertainties would be a potential future direction. Fourth, likelihood-based inference may suffer from local maxima [24]. Although we didn’t identify significant differences of different initiation points from our simulation study (more details in S1 Text), we recommend initiating the labels of genes from a known risk gene set or running with multiple starts for real data application (more details in S1 Text). Also, we observe the Gibbs sampler tends to move around local maxima for some time before convergence. Empirically, we suggest running at least 2,000 times of iterations and discarding the first 1,000 iterations as burn-ins. Fifth, we only considered the simulation verification under the ground truth model based on our assumptions, the generalizability to other alternative models is unexplored.

Sixth, to apply our model to other diseases, practitioners should be cautious if they would like to use the mutability of genes from a public dataset. 1) For WES data, the target region for each study could be different, which further results in differences in the calculation of mutability for the coding region 2) Mutability may be calculated based on a specific functional annotation of variants. Studies that use divergent classification criteria for variants should not share the same mutability. 3) Publicly available mutability may have been adjusted for cohort-specific parameters, such as sequencing depth, which may also affect the results if adapted to another cohort.

In addition, we only considered damaging DNVs and assumed the relative risk parameter *γ* is the same across all genes in N-DATA, which may cause our model to lose power if it varies across variants with different functions (e.g., LoF and Dmis). Future studies may explore adding functional annotation of variants as a layer in the model to further improve statistical power.

## Supporting information

S1 Text Supplementary notes on methods and results. Fig A in S1 Text: Power comparison of N-DATA w/o and w/ PPI network models. Fig B in S1 Text: FDR comparison of N-DATA w/o and w/ PPI network models. Fig C in S1 Text: FDR comparison of TADA-De novo, TADA-De novo p-values + DAWN and N-DATA. Fig D in S1 Text: Time comparison of TADA-De novo, TADA-De novo p-values + DAWN and N-DATA.
(DOCX)

S1 FigN-DATA identified 323 genes in network $G_{2}$.Green labels indicate the 18 genes identified when no network information was provided for N-DATA, and red labels indicate the additional 305 genes identified when the $G_{2}$ network was integrated. Circles indicate the 27 known human CHD genes, and squares indicate the 296 novel genes.(SVG)

S1 Table Simulation results. (XLSX)

S2 Table Results of real data application. (XLSX)
