# Supplementary material for: Network assisted analysis of de novo variants using protein-protein interaction information identified 46 candidate genes for congenital heart disease
Source: PLoS Genet. 2022 Jun 7;18(6):e1010252. doi: 10.1371/journal.pgen.1010252 (PMC9205499; doi:10.1371/journal.pgen.1010252)
Supplement: S1 Text — Fig A in S1 Text: Power comparison of N-DATA w/o and w/ PPI network models. Fig B in S1 Text: FDR comparison of N-DATA w/o and w/ PPI network models. Fig C in S1 Text: FDR comparison of TADA-De novo, TADA-De novo p-values + DAWN and N-DATA. Fig D in S1 Text: Time comparison of TADA-De novo, TADA-De novo p-values + DAWN and N-DATA. (DOCX) [file pgen.1010252.s001.docx]

**Supporting Information**

Table of Contents

[Comparisons of N-DATA Model with PPI Network and N-DATA Model w/o PPI Network 1](#_Toc102213307)

[Comparisons of TADA, DAWN, and N-DATA 4](#_Toc102213308)

[Convergence & Number of MCMCs 6](#_Toc102213309)

[Initiation Strategies 8](#_Toc102213310)

[Interpretation for Real Data Results 10](#_Toc102213311)

[Comparison of Using Different PPI Databases (HINT [1] and STRING [2]) 11](#_Toc102213312)

# **Comparisons of N-DATA Model with PPI Network and N-DATA Model w/o PPI Network**

Both models controlled FDR well under all the settings. N-DATA model with PPI network had much better power than the model without PPI network when the sample size and $\beta$ were both low. When the sample size and $\beta$ were larger, the power of N-DATA without PPI network improved as expected. The power of N-DATA model became better when $\tau_{1}$ increases, while the power of N-DATA model without the PPI network did not change much, which can be expected given this model did not consider the network information. (Fig A and Fig B)

**
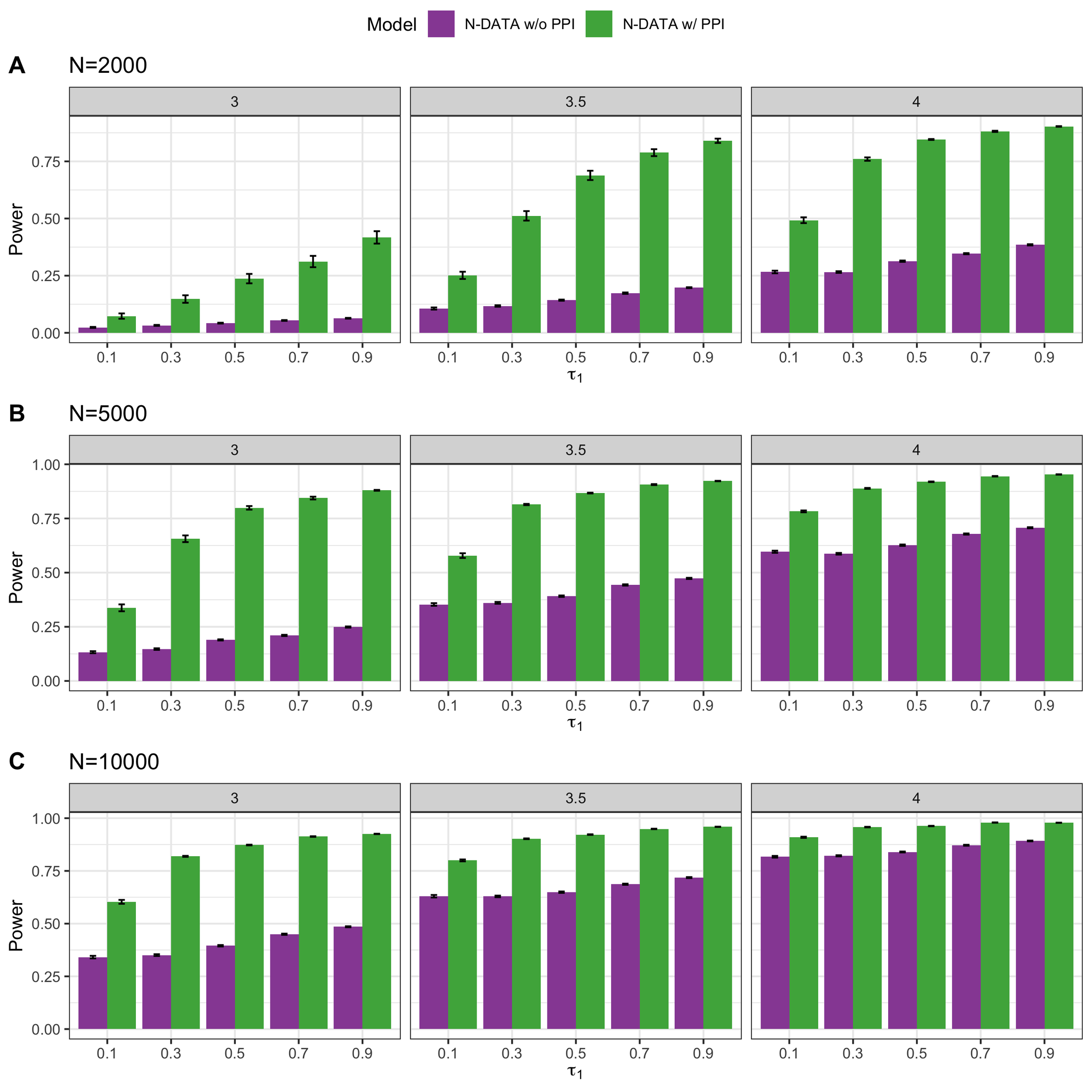
**

**Fig A**. **Power comparison of N-DATA w/o and w/ PPI network models**. Error bars represent standard errors estimated from 100 replications of simulation. Three panels in each sub-figure from left to right represent $\beta=3$, $\beta=3.5, \mathrm{and}$ $\beta=4,$respectively. Each panel shows the change of power when $\tau_{1}$ varies from 0.1 to 0.9. (A) Power comparison between the two models when the sample size is small ($N=2,000$). (B) Power comparison between the two models when the sample size is medium ($N=5,000$). (C) Power comparison between the two models when the sample size is large ($N=10,000$).

**
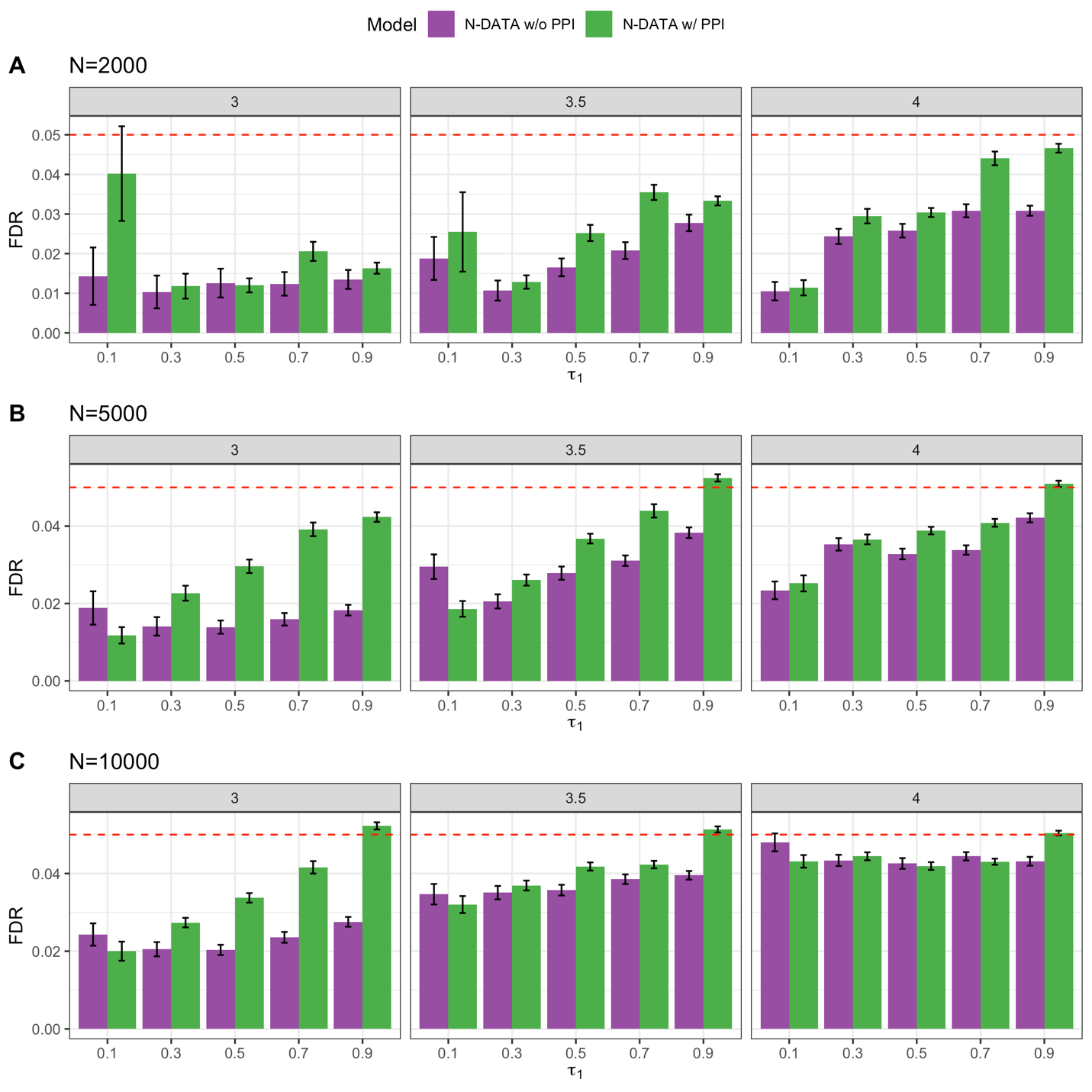
**

**Fig B**. **FDR comparison of N-DATA w/o and w/ PPI network models**. Error bars represent standard errors estimated from 100 replications of simulation. Three panels in each sub-figure from left to right represent $\beta=3$, $\beta=3.5, \mathrm{and}$ $\beta=4,$respectively. Each panel shows the change of FDR when $\tau_{1}$ varies from 0.1 to 0.9. (A) FDR comparison between the two models when the sample size is small ($N=2,000$). (B) FDR comparison between the two models when the sample size is medium ($N=5,000$). (C) FDR comparison between the two models when the sample size is large ($N=10,000$).

# **Comparisons of TADA, DAWN, and N-DATA**

**
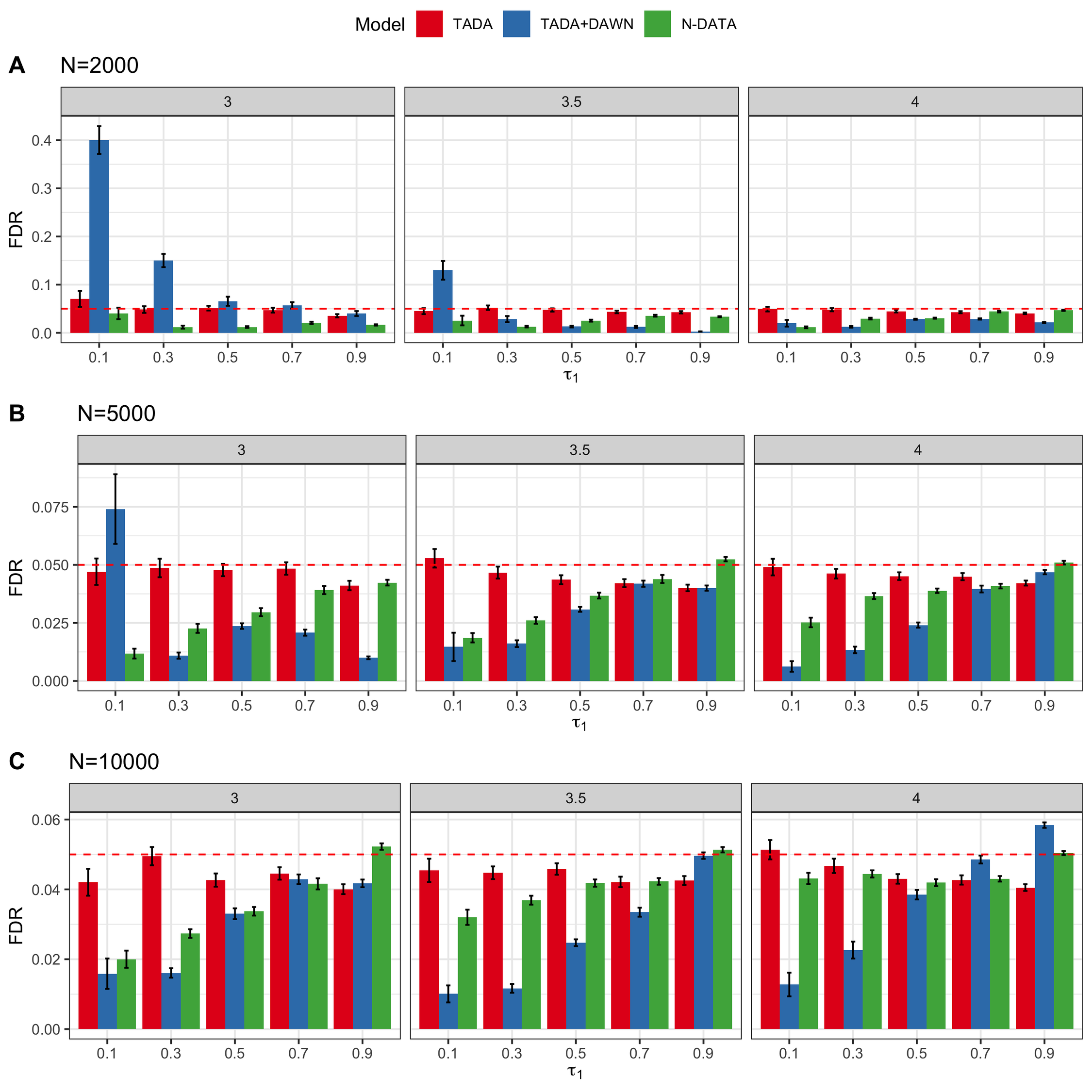
**

**Fig C**. **FDR** **comparison of TADA-*De novo*, TADA-*De novo* p-values + DAWN and N-DATA**. Error bars represent standard errors estimated from 100 replications of simulation. Three panels in each sub-figure from left to right represent $\beta=3$,$\beta=3.5, \mathrm{and}$ $\beta=4,$respectively. Each panel shows the change of FDR when $\tau_{1}$ varies from 0.1 to 0.9. (A) FDR comparison between the two models when the sample size is small ($N=2,000$). (B) FDR comparison between the three models when the sample size is medium ($N=5,000$). (C) FDR comparison between the three models when the sample size is large ($N=10,000$).

**
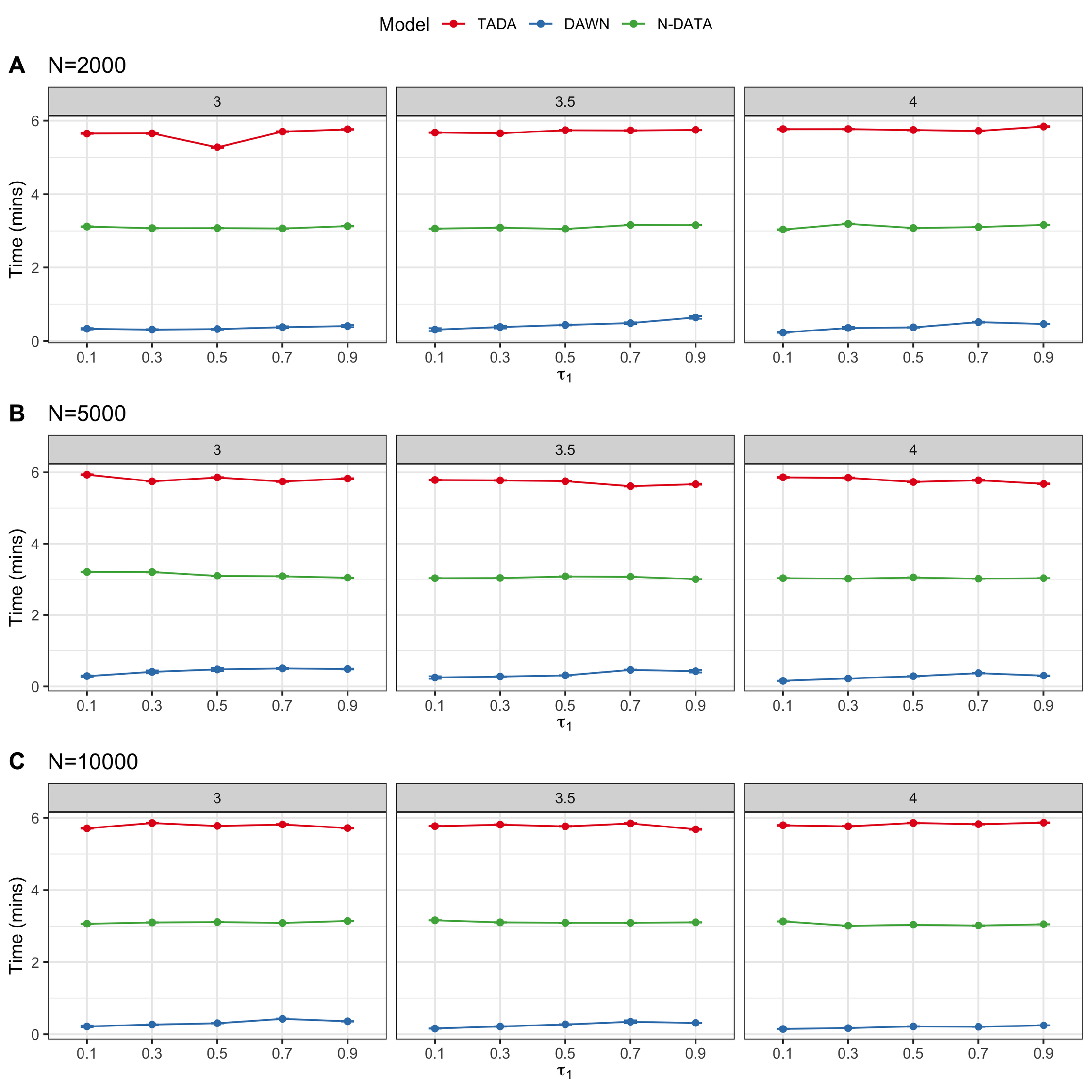
**

**Fig D**. **Time** **comparison of TADA-*De novo*, TADA-*De novo* p-values + DAWN and N-DATA**. Error bars represent standard errors estimated from 100 replications of simulation. Three panels in each sub-figure from left to right represent $\beta=3$,$\beta=3.5, \mathrm{and}$ $\beta=4,$respectively. Each panel shows the change of time when $\tau_{1}$ varies from 0.1 to 0.9. (A) Time comparison between the two models when the sample size is small ($N=2,000$). (B) Time comparison between the three models when the sample size is medium ($N=5,000$). (C) Time comparison between the three models when the sample size is large ($N=10,000$).

# **Convergence & Number of MCMCs**

We observed that we may suffer from convergence issue when the effect size $\beta$, the sample size $N$ or network parameter $\tau_{1}$ in $\theta_{0}$ is relatively low. The convergence issue occurs when during an iteration, no gene has the label of $S_{i}$=1 during the iteration, and the algorithm fails to converge. We have added more simulation settings to illustrate this issue. We varied sample size $N$ at 1000 and 2000, effect size $\beta$ at 2.5, 3 and 3.5, and $\tau_{1}$ at 0.1, 0.3, 0.5, 0.7 and 0.9, and reported the success rate of not meeting any convergence issue during 100 simulation replications.

| $N=1000$ | # of risk genes | $\beta=2.5$ | $\beta=3$ | $\beta=3.5$ |
| --- | --- | --- | --- | --- |
| $\theta_{0}=(-4,0.1,0)$ | 67 | 50% | 71% | 98% |
| $\theta_{0}=(-4,0.3,0)$ | 157 | 68% | 97% | 100% |
| $\theta_{0}=(-4,0.5,0)$ | 228 | 80% | 100% | 100% |
| $\theta_{0}=(-4,0.7,0)$ | 283 | 98% | 99% | 100% |
| $\theta_{0}=(-4,0.9,0)$ | 353 | 98% | 100% | 100% |
| $N=2000$ | # of risk genes | $\beta=2.5$ | $\beta=3$ | $\beta=3.5$ |
| $\theta_{0}=(-4,0.1,0)$ | 67 | 85% | 99% | 100% |
| $\theta_{0}=(-4,0.3,0)$ | 157 | 97% | 100% | 100% |
| $\theta_{0}=(-4,0.5,0)$ | 228 | 100% | 100% | 100% |
| $\theta_{0}=(-4,0.7,0)$ | 283 | 100% | 100% | 100% |
| $\theta_{0}=(-4,0.9,0)$ | 353 | 100% | 100% | 100% |

**Table A. Success rate of N-DATA algorithm under settings of small** $\boldsymbol{N}$**,** $\boldsymbol{\beta}$ **or** $\boldsymbol{\tau}_{\boldsymbol{1}}$**.**

We compared the power and FDR results under different simulation settings with 5,000 MCMC iterations with the first 2,000 as burn-ins versus 2,000 MCMC iterations and 1,000 burn-ins when sample size $N$ is 2,000. From the figure below, we observed that the number of MCMC iterations had limited influence on the power and FDR in our settings.

**
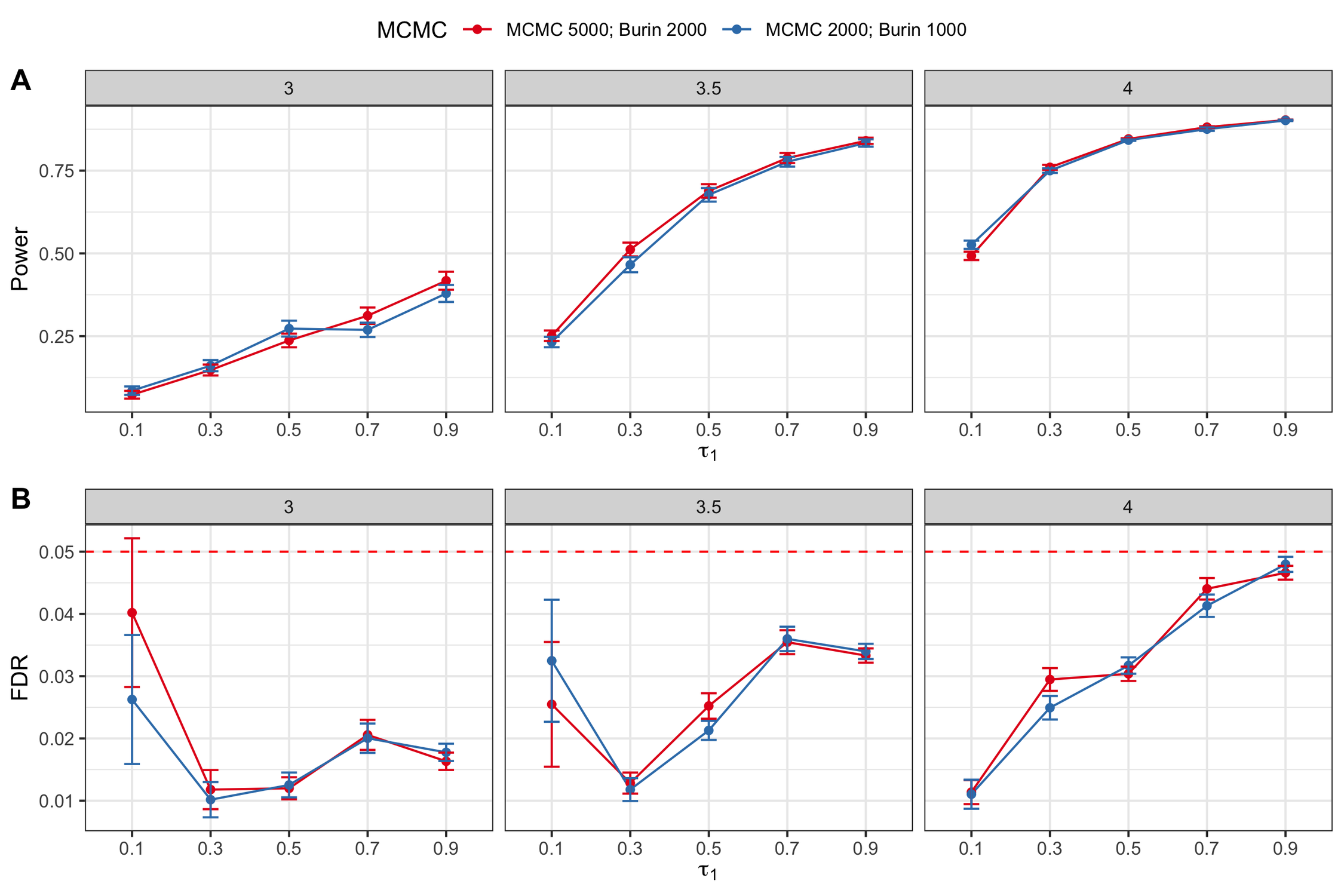
**

**Fig E**. **Power and FDR** **comparison of different MCMC and burn-in settings**. Error bars represent standard errors estimated from 100 replications of simulation. Three panels in each sub-figure from left to right represent $\beta=3$,$\beta=3.5, \mathrm{and}$ $\beta=4,$respectively. The sample size $N$ is 2,000. The red line represents 5,000 MCMC iterations with 2,000 burn-ins, and the blue line represents 2,000 MCMC iterations with 1,000 burn-ins. (A) Power comparison of Different MCMC and Burn-in Settings when $\tau_{1}$ varies from 0.1 to 0.9. (B) FDR comparison of Different MCMC and Burn-in Settings when $\tau_{1}$ varies from 0.1 to 0.9.

# **Initiation Strategies**

**
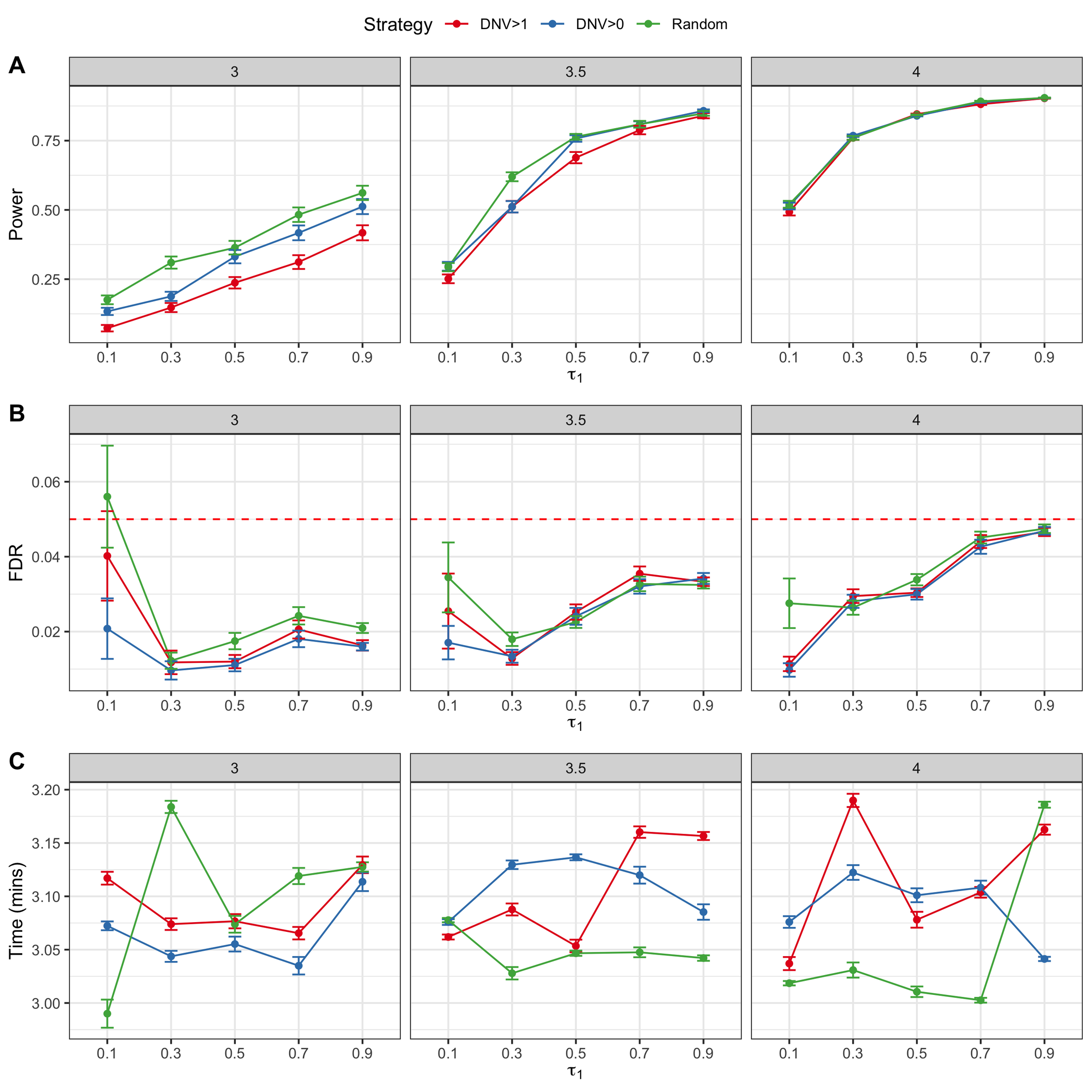
**

**Fig F**. **Power, FDR, and time comparison under different initiation strategies when** $\boldsymbol{N=2,000}$. Error bars represent standard errors estimated from 100 replications of simulation. Three panels in each sub-figure from left to right represent $\beta=3$,$\beta=3.5, \mathrm{and}$ $\beta=4,$respectively. (A) Power comparison under different initiation strategies when $\tau_{1}$ varies from 0.1 to 0.9. (B) FDR comparison under different initiation strategies when $\tau_{1}$ varies from 0.1 to 0.9. (C) Time comparison under different initiation strategies when $\tau_{1}$ varies from 0.1 to 0.9.

**
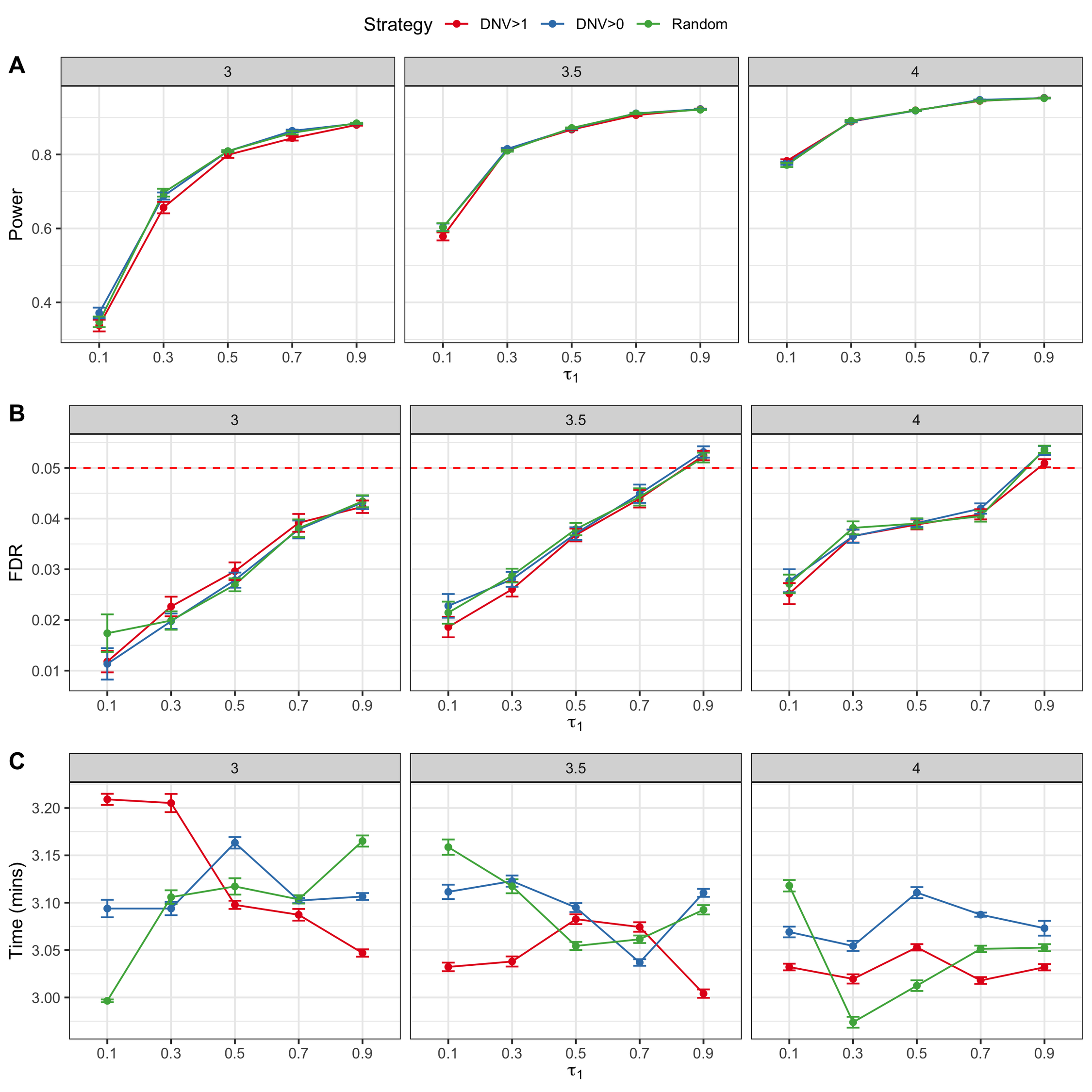
**

**Fig G**. **Power, FDR, and time comparison under different initiation strategies when** $\boldsymbol{N=5,000}$. Error bars represent standard errors estimated from 100 replications of simulation. Three panels in each sub-figure from left to right represent $\beta=3$,$\beta=3.5, \mathrm{and}$ $\beta=4,$respectively. (A) Power comparison under different initiation strategies when $\tau_{1}$ varies from 0.1 to 0.9. (B) FDR comparison under different initiation strategies when $\tau_{1}$ varies from 0.1 to 0.9. (C) Time comparison under different initiation strategies when $\tau_{1}$ varies from 0.1 to 0.9.

# **Interpretation for Real Data Results**

In our simulation study, we also observed that under the ground truth model, there were a lot of true risk genes with 0 counts. For instance, when there are 353 true risk genes ($\tau_{1}=0.9$) in the network, there are about 200 genes without DNV when $\beta=3.5$and $N=2,000.$ Under this scenario, N-DATA can identify about 150 genes with 0 counts that are true risk genes. This is in line with our real data observation. For the real data, although some of the top genes identified did not have DNVs, they were genes with many neighbors in the PPI network (Fig G). The active interactions with other genes lifted their probability of being true risk genes, resulting in low q-values in the model (Fig H).

**
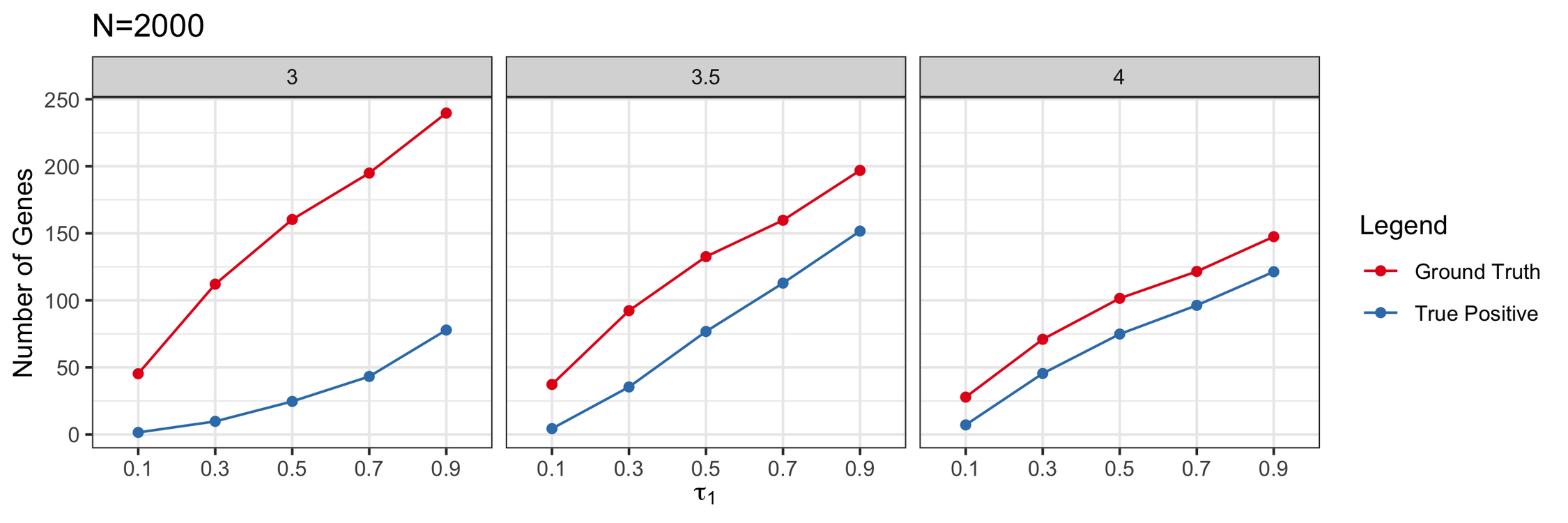
**

**Fig H**. **Number of risk genes in the ground truth model and number of true risk genes identified by N-DATA when** $\boldsymbol{N=2,000}$. Three panels from left to right represent $\beta=3$,$\beta=3.5, \mathrm{and}$ $\beta=4,$respectively. Each panel shows the change of numbers when $\tau_{1}$ varies from 0.1 to 0.9.

**
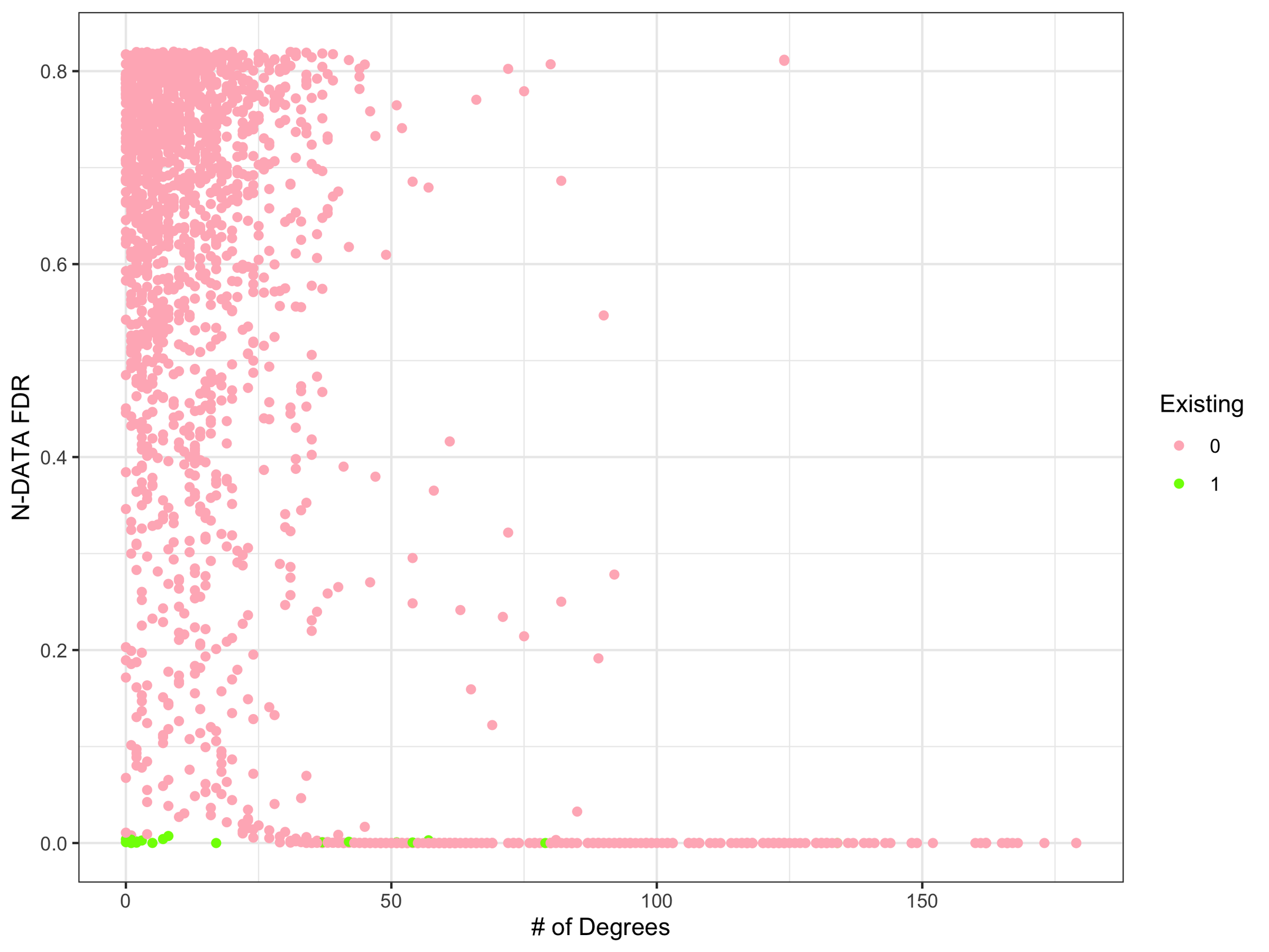
**

**Fig I**. **Scatter plot for N-DATA FDR q-values (y-axis) versus number of degrees in network** $\mathcal{G}_{\boldsymbol{2}}$ **(x-axis)**. Green dots represent genes that can be identified by N-DATA w/o network model, and pink dots represent additional genes can be identified by N-DATA after integrating network $\mathcal{G}_{2}$.

# **Comparison of Using Different PPI Databases (HINT [1] and STRING [2])**

To investigate the performance with N-DATA on different PPI networks, we integrated the network from HINT with DNVs from CHD to conduct further analysis. We downloaded the high-quality binary and co-complex interactions (updated in August 2021) from <http://hint.yulab.org/download/>. In order to build the network with reliable edges, we screened interactions being reported by at least 4 independent publications for all interactions. Then, we used the same procedure to construct the PPI network for CHD as we did for the STRING database. A comparison of constructed network $\mathcal{G}_{2}$ based on STRING and HINT databases is summarized in the table below.

|  | Number of Genes | Number of Edges |
| --- | --- | --- |
| HINT | 1,785 | 16,668 |
| STRING | 1,814 | 21,468 |
| Overlap | 679 | 1,834 |

**Table B. Comparison between network** $\mathcal{G}_{\boldsymbol{2}}$ **built from HINT and STRING**

As can be seen, the number of overlapping edges is small compared to the total number of edges. Then, we applied N-DATA on the constructed HINT PPI network with CHD DNV data. In Fig I, we visualized all genes included in the analysis, and significant genes identified with HINT and STRING, respectively. Overall, we observed that the output from N-DATA depended on the choice of the PPI database. Using different PPI networks and different filtering criteria could result in a different set of significant genes. However, even the majority of identified genes were different when adopting different PPI databases, there could be shared biological insights learning from considering different PPI databases as discussed in the following.

We tested the significance of the overlap between genes identified in the two networks (only overlapping genes for HINT network $\mathcal{G}_{2}$ and STRING network $\mathcal{G}_{2}$ were considered as background genes) through Fisher’s Exact test, and found a significant association between the genes identified from HINT and STRING (p=3.14e-12). Further, we conducted functional enrichment analysis for the 25 overlapping genes identified from the two databases in Human Phenotype Ontology gene lists using g:Profiler [3]. The top five HPO [4] terms were atrial septal defect (HP:0001631), abnormal atrial septum morphology (HP:0011994), abnormal cardia atrium morphology (HP:0005120), abnormal morphology of the great vessels (HP:0030962), and abnormal pulmonary valve morphology (HP:001641), which are subtypes of CHD. In addition, we conducted a pathway enrichment analysis for the identified gene lists from HINT and STRING using Ingenuity Pathways Analysis (IPA, QIAGEN Inc.) [5], respectively. We visualized the correlation of p-values output from 277 overlapping pathways in IPA (Fig J). The Pearson’s correlation was 0.48, and the correlation was significant (p<2.2e-16).


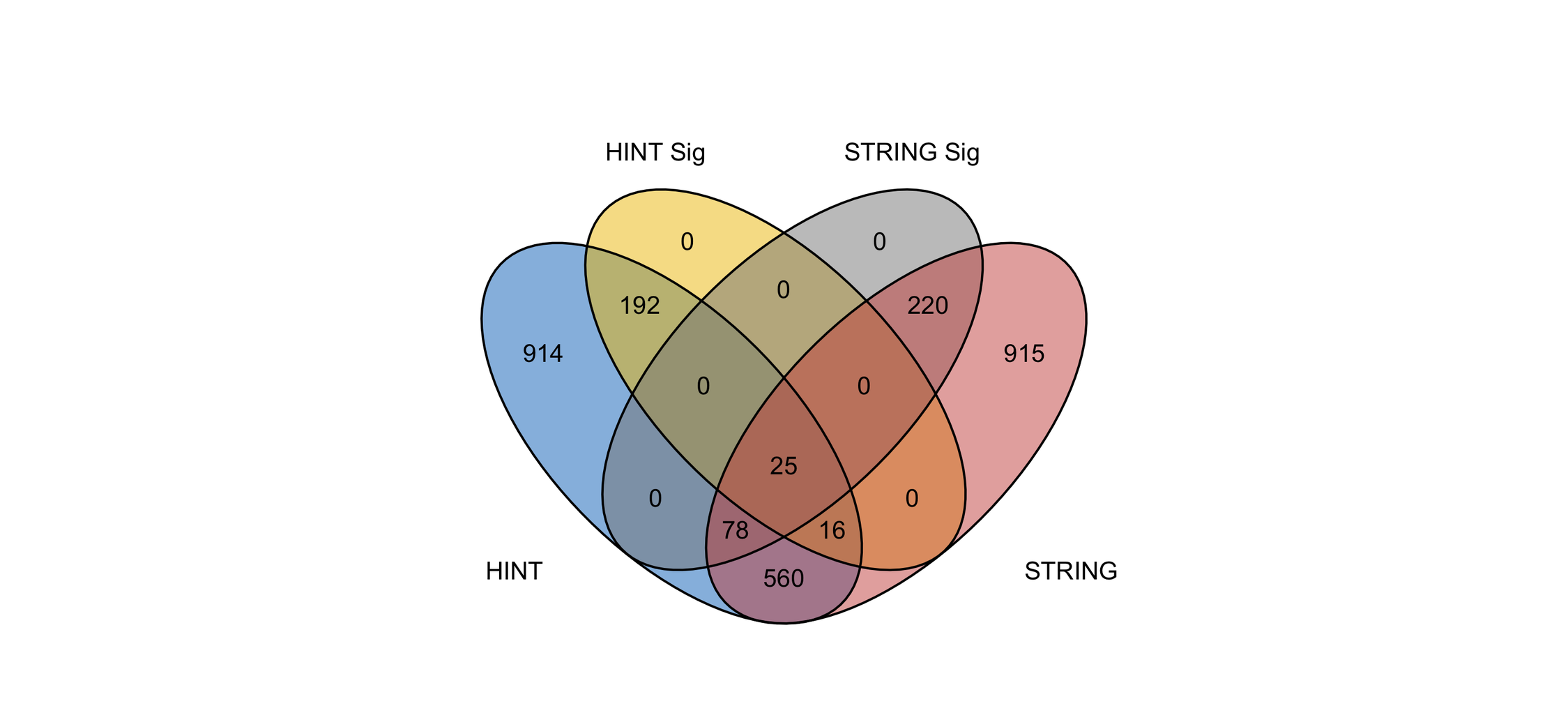


**Fig J. Venn diagram of genes included in the analysis using HINT network, significant genes identified by N-DATA using HINT, genes included in the analysis using STRING network, and significant genes identified by N-DATA using STRING.**

**
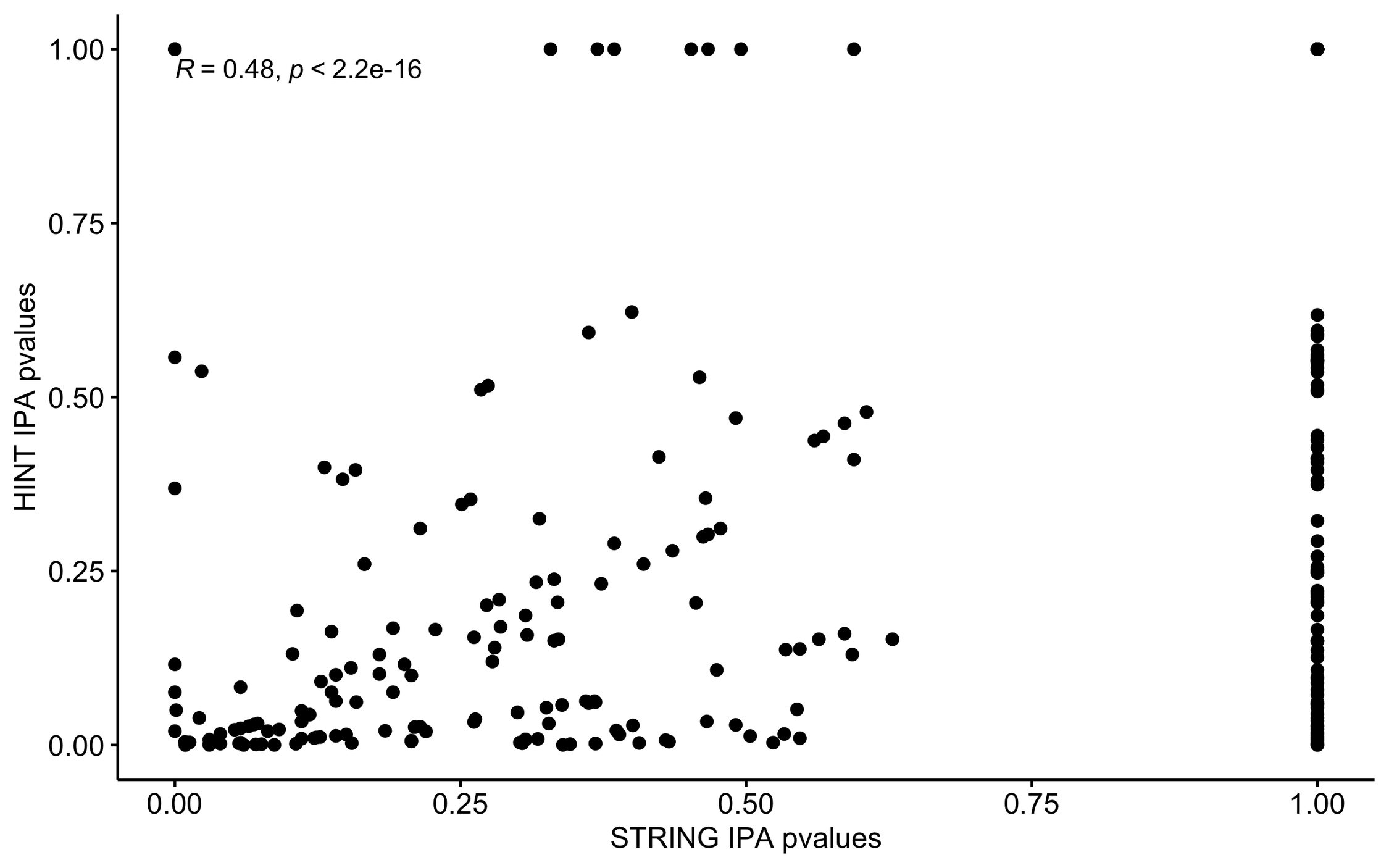
**

**Fig K. Scatter plot of p-values from 277 overlapping pathways from the output of IPA.** X-axis represents p-values of each pathway using 323 significant genes identified from STRING, and y-axis represents p-values of each pathway using 233 significant genes identified from HINT.

**Reference**

1. Das J, Yu H. HINT: High-quality protein interactomes and their applications in understanding human disease. BMC Systems Biology. 2012;6(1):92. doi: 10.1186/1752-0509-6-92.

2. Szklarczyk D, Gable AL, Lyon D, Junge A, Wyder S, Huerta-Cepas J, et al. STRING v11: protein-protein association networks with increased coverage, supporting functional discovery in genome-wide experimental datasets. Nucleic Acids Res. 2019;47(D1):D607-d13. Epub 2018/11/27. doi: 10.1093/nar/gky1131. PubMed PMID: 30476243; PubMed Central PMCID: PMCPMC6323986.

3. Reimand J, Arak T, Adler P, Kolberg L, Reisberg S, Peterson H, et al. g:Profiler-a web server for functional interpretation of gene lists (2016 update). Nucleic acids research. 2016;44(W1):W83-W9. Epub 2016/04/20. doi: 10.1093/nar/gkw199. PubMed PMID: 27098042.

4. Köhler S, Gargano M, Matentzoglu N, Carmody LC, Lewis-Smith D, Vasilevsky NA, et al. The Human Phenotype Ontology in 2021. Nucleic Acids Res. 2021;49(D1):D1207-d17. Epub 2020/12/03. doi: 10.1093/nar/gkaa1043. PubMed PMID: 33264411; PubMed Central PMCID: PMCPMC7778952.

5. Krämer A, Green J, Pollard J, Jr, Tugendreich S. Causal analysis approaches in Ingenuity Pathway Analysis. Bioinformatics. 2013;30(4):523-30. doi: 10.1093/bioinformatics/btt703.
